# Differential impacts of land‐use change on multiple components of common milkweed (*Asclepias syriaca*) pollination success

**DOI:** 10.1002/ece3.11494

**Published:** 2024-06-06

**Authors:** David J. Rockow, Carlos Martel, Gerardo Arceo‐Gómez

**Affiliations:** ^1^ Department of Biological Sciences East Tennessee State University Johnson City Tennessee USA; ^2^ Royal Botanic Gardens, Kew Richmond UK

**Keywords:** disturbance, land‐use change, milkweed, pollen transfer, pollination

## Abstract

Land‐use change is one the greatest threats to biodiversity and is projected to increase in magnitude in the coming years, stressing the importance of better understanding how land‐use change may affect vital ecosystem services, such as pollination. Past studies on the impact of land‐use change have largely focused on only one aspect of the pollination process (e.g., pollinator composition, pollinator visitation, and pollen transfer), potentially misrepresenting the full complexity of land‐use effects on pollination services. Evaluating the impacts across multiple components of the pollination process can also help pinpoint the underlying mechanisms driving land‐use change effects. This study evaluates how land‐use change affects multiple aspects of the pollination process in common milkweed populations, including pollinator community composition, pollinator visitation rate, pollen removal, and pollen deposition. Overall, land‐use change altered floral visitor composition, with small bees having a larger presence in developed areas. Insect visitation rate and pollen removal were also higher in more developed areas, perhaps suggesting a positive impact of land‐use change. However, pollen deposition did not differ between developed and undeveloped sites. Our findings highlight the complexity evaluating land‐use change effects on pollination, as these likely depend on the specific aspect of pollination evaluated and on the of the intensity of disturbance. Our study stresses the importance of evaluating multiple components of the pollination process in order to fully understand overall effects and mechanisms underlying land‐use change effects on this vital ecosystem service.

## Graphical abstract

This study evaluates how urbanization affects multiple aspects of the pollination process in common milkweed populations, including community composition, pollinator visitation rate, pollen removal, and deposition on stigmas. We found differential effects of urbanization on the varied components of pollination, hence highlighting the complexity of evaluating overall urbanization effects on pollination.

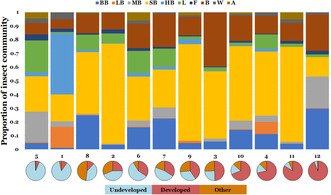

## 1 INTRODUCTION

Nearly all contemporary environmental threats are directly related to human activities. Land‐use change, the conversion of natural habitat to areas fit for human exploitation, is thought to be the most detrimental anthropogenic stressor and has widely been implicated in declines in species richness and abundance across many taxonomic groups (e.g., Davison et al., 2021; Diaz et al., 2019; Sala et al., 2000). Land‐use change is especially harmful because it degrades existing habitat, while making adjacent natural areas further exposed to human impacts (i.e., pollution, pesticide usage, and invasive species) and increasingly fragmented (Haddad et al., 2015). About 70% of terrestrial ecoregions are considered moderately or highly modified by human activity, while just ~5% exhibit no anthropogenic modification (Kennedy et al., 2019). With anthropogenic disturbances only projected to increase in magnitude, it is becoming increasingly important to evaluate the potential negative impacts that human disturbances, particularly land‐use change, can have on vital ecosystem functions and services.

One important ecosystem service is pollination. Approximately 87.5% of flowering plants are animal pollinated (Ollerton et al., 2011), with fruit/seed yield of ~70% of crop plants positively impacted by animal pollination (Klein et al., 2007). In fact, recent studies suggest that the reproduction of at least half of all flowering species would experience an 80% decline without pollinators (Rodger et al., 2021). Currently, pollinators are under global decline due to a variety of anthropogenic stressors, including invasive species spread, pesticide usage, climate change, and land‐use change (González‐Varo et al., 2013; Potts, Imperatriz‐Fonseca, Ngo, Aizen, et al., 2016; Stout & Dicks, 2022; Vanbergen & the Insect Pollinators Initiative, 2013). Recent estimates indicate roughly 40% of insect pollinator species are threatened with local extinction (Potts, Imperatriz‐Fonseca, Ngo, Biesmeijer, et al., 2016). The ecologic and economic importance of pollination, coupled with the growing threat of land‐use change, makes it imperative to better understand how pollination success can be impacted by human activities. So far, studies on the impact of land‐use change on the pollination process have often reached different conclusions (Rivkin et al., 2020). While some studies find land‐use change negatively impacts pollination success (Bennett et al., 2020; Winfree et al., 2011), others find a negligible or even positive impacts (Theodorou et al., 2016; Wenzel et al., 2020; Winfree et al., 2007). One potential underlying cause for this discordance is that studies typically focus on a single aspect of pollination success, whether that be changes in pollinator community (e.g., Deguines et al., 2016) or pollen transport (e.g., Bennett et al., 2020). Land‐use change, however, may induce differential impacts across different stages of the pollination process. Hence, focusing on one pollination aspect may limit our understanding of the full consequences and the underlying mechanisms by which land‐use change affects pollination success.

Changes in pollinator abundance are the most studied aspect of pollination in studies that evaluate the effects of land‐use change. As a result, there is substantial evidence that land‐use change negatively impacts pollinator abundance (Desaegher et al., 2018; Fortel et al., 2014; Kremen et al., 2002; Liang et al., 2023; Millard et al., 2021; Winfree et al., 2011). For instance, a meta‐analysis on the impacts of land‐use change on pollination found strong decreases in pollinator richness and abundance, with the magnitude of pollinator declines increasing with increased severity of land‐use change (Winfree et al., 2011). Though even moderate levels of urbanization have been found to significantly reduce richness and abundance across multiple pollinator taxa (Desaegher et al., 2018), heavily urbanized areas typically have lower abundance of floral resources, and a higher percent of impervious surfaces (decreased nesting area), both of which negatively impact pollinator abundance (Baldock, 2020). Interestingly, a few studies have found the opposite effect with moderately urbanized areas having higher pollinator abundance, when compared to undisturbed sites (Biella et al., 2022; Carper et al., 2014; Sing et al., 2016; Theodorou et al., 2016; Udy et al., 2020; Wenzel et al., 2020). Additionally, urban gardens have been found to host a large diversity of pollinators, even in heavily developed environments (Frankie et al., 2005; Matteson et al., 2008), further emphasizing the role of floral resources and non‐impervious surfaces in positively maintaining pollinator abundance.

Land‐use changes have been shown to variably impact pollinator taxa, resulting in changes in pollinator community composition (Cane et al., 2006; Geslin et al., 2013; Udy et al., 2020). A study on four insect orders (Hymenoptera, Lepidoptera, Coleoptera, and Diptera) across an urban gradient found that the species richness for each group was differentially impacted by urbanization, with Hymenoptera being the most resilient to urbanization and Lepidoptera being the most sensitive (Deguines et al., 2016). Additionally, generalist pollinators have been shown to be more resilient to land‐use change than specialists (Deguines et al., 2016; Winfree et al., 2011). Pollinator community composition can be an important mediator of plant reproductive success because pollinator species can vary in the quantity and quality of pollen they remove and deposit, due to differences in body size, foraging behavior, and degree of specialization (Cullen et al., 2021; Kandori, 2002; Sahli & Conner, 2007). Thus, changes in pollinator community composition can have differential impacts on pollen removal and pollen deposition (Thomson & Goodell, 2001; Wilson & Thomson, 1991). The impacts of urbanization on pollen removal (Breitbart et al., 2022; Ushimaru et al., 2014) and deposition (Carper et al., 2014) have often been studied separately. One of the few studies that evaluated pollen removal and deposition across an urbanization gradient found no effect of land‐use, though the study was on a specialized hawkmoth pollinated flower (Skogen et al., 2016). Such studies in more generalized pollination systems are lacking. To our knowledge, little work has been done comparing the overall effectiveness of diverse pollinator communities in developed versus undeveloped habitats. Evaluating patterns of pollen removal and deposition across a land‐use change gradient, in conjuncture with changes in pollinator visitation rate and community composition, has the potential to advance our understanding of the impact of land‐use change on this key ecosystem service. Evaluating effects on multiple aspects of the pollination process will also help pinpoint the underlying mechanisms driving the impacts of land‐use change on pollination. Such knowledge will help inform decisions that aim to conserve the integrity of pollination in anthropogenically disturbed environments.

Furthermore, studies that evaluate the impact of land‐use changes on pollination success have predominately looked at “traditional” pollination systems where pollen is presented as individual grains. More derived pollination systems, such as in milkweeds, where pollen is stored in discrete packets have been less studied. Milkweeds are an ideal system to evaluate the impacts of land‐use change on pollen removal and deposition because the large pollen packets are easily visible and quantifiable in the field. Additionally, pollen packets are unique in size and shape for each milkweed species (Wyatt & Broyles, 1994), eliminating potentially complicating factors such as heterospecific pollen receipt (Ashman & Arceo‐Gómez, 2013). Furthermore, despite its morphological specialization, common milkweed is a pollination generalist. Thus, findings may advance our understanding of the effects of land‐use change on the pollination of species across the specialization‐generalization continuum. Due its high degree of floral visitor generalization, we expect land‐use change to have a small effect, or perhaps even a positive effect, on pollinator visitation rate to milkweed flowers. However, land‐use change has been shown to differentially impact pollinator taxa (Cane et al., 2006; Geslin et al., 2013; Udy et al., 2020), and thus, we expect pollinator community composition to differ between developed and undeveloped areas. For instance, we could expect land‐use change to lead to an increase in the abundance of Hymenopterans and a lower abundance of Lepidopterans (e.g., Deguines et al., 2016). Land‐use change has also been shown to decrease visitation of the most efficient pollinators (e.g., Deguines et al., 2016; Winfree et al., 2011). Hence, we could also expect changes in patterns of pollen removal and deposition and in developed compared to undeveloped areas. Common milkweed is of high conservation importance due to its integral role in the monarch butterfly (*Danaus plexippus*) lifecycle, serving as the oviposition site, and later as a food source for monarch caterpillars (Baker & Potter, 2018; Pocius et al., 2018). Planting milkweed in developed areas is a widespread conservation strategy currently employed to bolster monarch numbers (Baker & Potter, 2018). Thus, studying how pollination success for common milkweed is impacted by land‐use changes can further aid in developing strategies to best maintain urban populations of common milkweed, indirectly aiding in monarch conservation efforts.

Overall, in this study we aim to (1) evaluate differences in pollinator visitation rate and community composition and to (2) assess changes in the amount of pollen removed and deposited on milkweed flowers between populations in developed and undeveloped areas.

## 2 METHODS

### 2.1 Study species

Common milkweed (*Asclepias syriaca*, Apocynaceae) is a widespread species native to the Eastern United States and Canada. Common milkweed occurs in a variety of both developed and undeveloped habitats, including roadsides, vacant lots, pastures, prairies, and forest edges with high levels of sun light, making it an ideal study system to evaluate pollination success in a diverse array of habitats. Common milkweed plants produce an average of 4.3 inflorescences with 83.6 pink to white flowers per inflorescence, though these figures are highly variable (Betz & Lamp, 1990; counts in the field ranged from 1–12 infloresences per plant and 10–140 flowers per infloresence). Each flower typically has five sepals and five petals, as well as a showy corona made up of five hoods, each containing a fragrant nectar pool (Wyatt & Broyles, 1994). On either side of each floral hood is a stigmatic slit (five total), which contains one pollinarium. Each pollinarium consists of two pollen packets, or pollinia, connected via a central corpusculum. Each pollinia contains hundreds of pollen grains (Wyatt & Broyles, 1994).

Due to its unique pollen morphology, common milkweed has a specialized pollination mechanism, while foraging for nectar a pollinator will insert an appendage into a stigmatic slit, thus contacting the corpusculum (Woodson, 1954; Wyatt & Broyles, 1994). The corpusculum is grooved and adheres to the pollinator, so when the insect frees itself it will remove the entire pollinarium from the stigmatic slit (Woodson, 1954; Wyatt & Broyles, 1994). The pollinator may then insert the pollinarium‐attached appendage into a stigmatic slit and deposit one pollinium (Woodson, 1954; Wyatt & Broyles, 1994).

Despite its specialized pollination mechanism, common milkweed attracts a wide diversity of pollinators across four insect orders: Hymenoptera (bees, wasps, and ants), Lepidoptera (butterflies and moths), Coleoptera (beetles), and Diptera (flies) (Howard & Barrows, 2014). However, the majority of studies on common milkweed pollinators have identified either bumblebees or honeybees as the main pollinators (Jennersten & Morse, 1991; Kephart & Theiss, 2004; MacIvor et al., 2017; Morse, 1981; Stevens, 1951; Willson & Bertin, 1979). Other studies have also identified flies, wasps, soldier beetles (Cantharidae), moths, and butterflies as effective pollinators of common milkweed (Gustafson et al., 2023; Howard & Barrows, 2014; Kephart & Theiss, 2004; Stevens, 1951; Willson & Bertin, 1979).

### 2.2 Study sites

**FIGURE 1:**
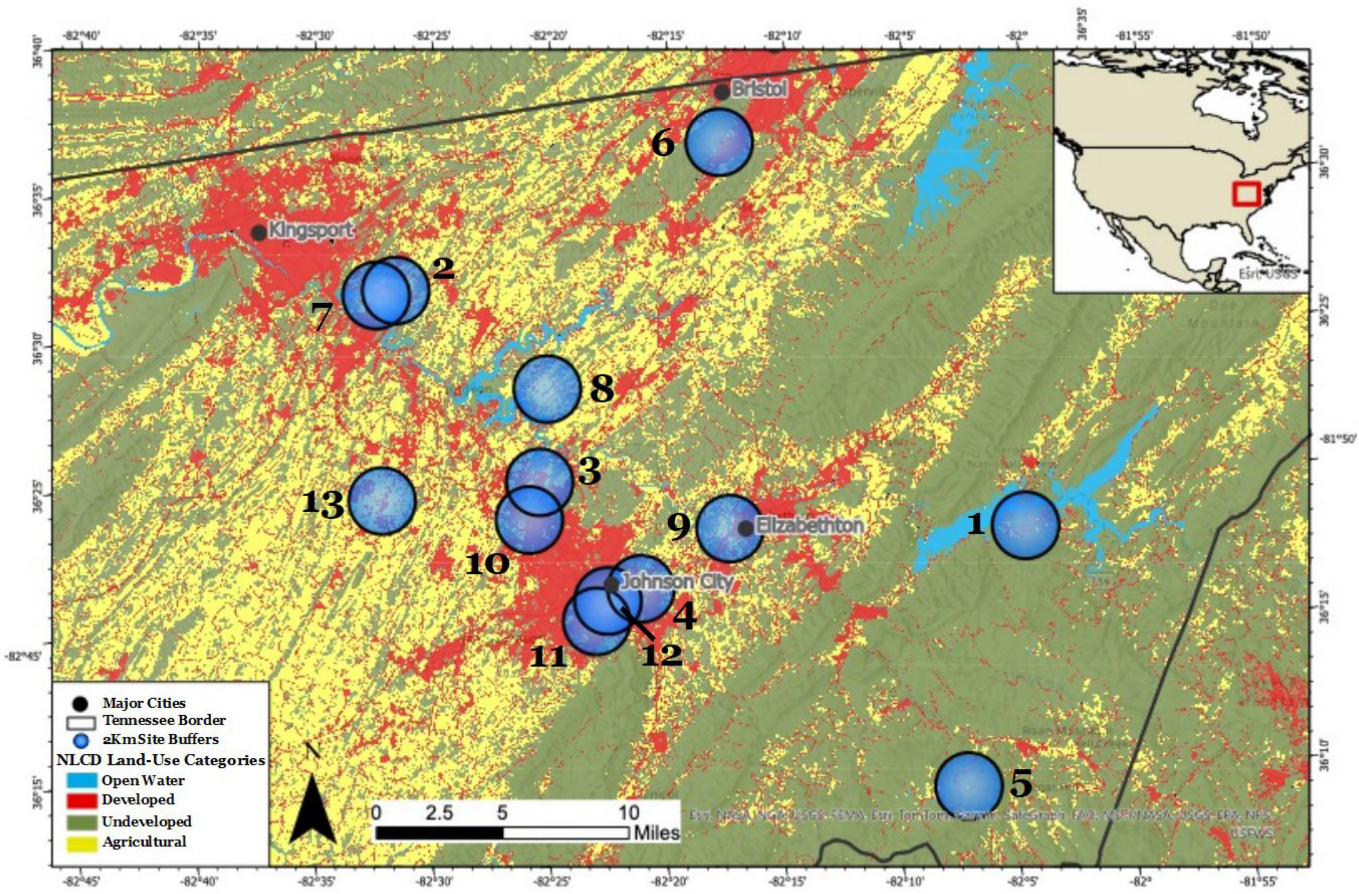
Location of surveyed milkweed populations mapped on a land‐use map. The black outline represents the Tennessee border, and map on the upper‐right corner map shows the general location within the United States. Site 13 was not included in analyses due to a lack of sufficient data.

Data were collected at 13 common milkweed populations in the Tri‐Cities region of Northeast Tennessee, USA. The Tri‐Cities region incorporates the cities of Bristol, Kingsport, and Johnson City, with the area between each city comprised mainly of suburban sprawl, pastureland, and deciduous forest (Figure 1). Data were collected during the summers of 2021 and 2022. Details regarding exact location, population size, and other attributes of each site are provided in Table A1. Two of the populations were managed in gardens (sites 9 and 12), while the rest were naturally occurring (not planted) populations.

### 2.3 Land‐use classification

Land‐use around each site was quantified by mapping out the sites in ArcGIS (2.9) and determining the land‐use within a two‐kilometer buffer around each site. While most bee species have a maximum foraging distance of less than 1 km, some species, including honeybees and bumblebees, can forage across distances of 10+ kilometers (Grüter & Hayes, 2022). Two kilometers was used as the land‐use buffer because it encompassed the foraging range of most bees, while also accounting for the larger foraging radii of honeybees and bumblebees, which are typically the most abundant milkweed pollinators (Jennersten & Morse, 1991; Kephart & Theiss, 2004; MacIvor et al., 2017; Morse, 1981; Stevens, 1951; Willson & Bertin, 1979).

We used land‐use definitions as designated by the 2019 National Land Cover Database (NLCD) (Dewitz, 2021), a project of the Multi‐Resolution Land Characteristics Consortium (MRLC) that utilizes Landsat satellite imagery data to produce comprehensive land‐cover maps. Specifically, the NLCD employs a modified Anderson II Classification System to categorize land surface into discrete categories using factors such as vegetation type, percent tree cover, and percent impervious surface cover. With 30‐m resolution NLCD data, the number of pixels per each land‐use category in the 2 km buffers was recorded as in past studies (Pozzi & Small, 2005; Watson et al., 2022) (see Table A2 for a percent breakdown of each land‐use type within a 2 km radius of each milkweed population). Fifteen unique NLCD land‐use categories were present within the 2 km buffers around the milkweed sites (Table A2), and these were subsetted into two major categories of land‐use: developed land and undeveloped land.

The NLCD defines developed land as any area that has been modified primarily for the purpose of anthropogenic utilization, excluding land primarily used for cultivation. The NLCD further separates developed space into four different categories based on the intensity of development (i.e., percent of impervious surfaces), which range from “Developed, Open Space” (<20% impervious surfaces) to “Developed, High Intensity” (>80% impervious surfaces).

Undeveloped NLCD land‐use categories include forest habitats, grasslands, shrublands, and wetlands.

**FIGURE 2:**
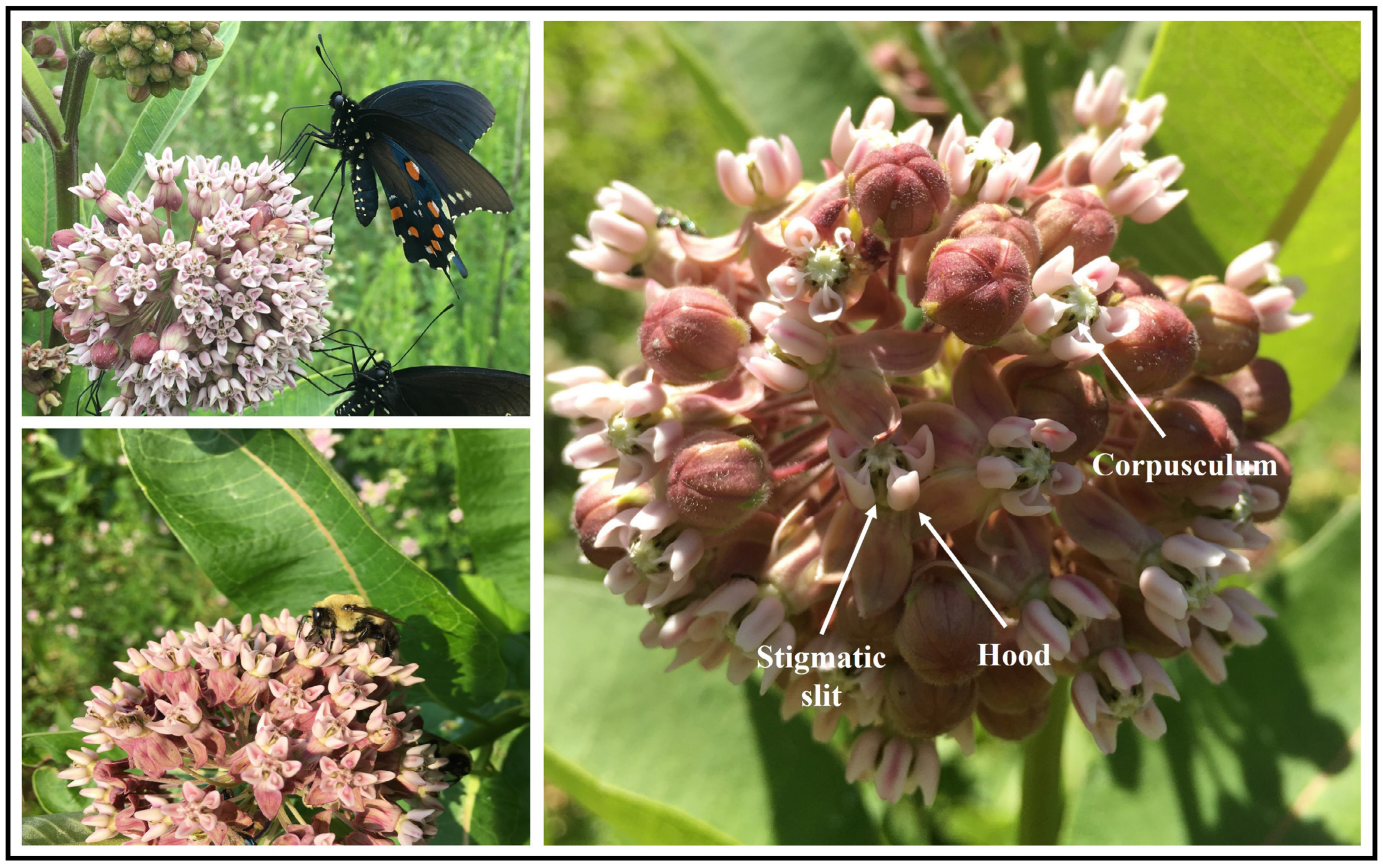
Photograph of common milkweed flowers showing the corpusculum, stigmatic slit and floral hood. To the left Milkwed flowers being visited by butterflies and bees.

Ultimately, sites with ≥40% undeveloped area (mean 60%, range: 41%–87%) were classified as undeveloped (sites 1–2 and 5–8), while sites with ≤33% undeveloped area (mean 17.3%, range 5%–33%) were classified as developed (sites 3–4 and 9–12; Figure 2). Both categories contained six sites total: two surveyed in 2021 and four surveyed in 2022 (Table A1).

### 2.4 Insect visitation rates and community composition

Each site was visited every other day during the duration of the flowering season (between June 17 and August 8). During each site visit, an insect survey was conducted (between four and fourteen surveys per site due to variation in flowering duration). Total survey time ranged between 120 and 435 min per site (Table A1). Insect surveys were conducted when at least five inflorescences were blooming on warm non‐rainy days. All surveys were conducted between 9:00 and 18:00, reflecting peak visitation for the primarily diurnally pollinated common milkweed (Jennersten & Morse, 1991; Morse & Fritz, 1983). Temperature at the time of the survey spanned from 18°C to 32°C. Every insect that landed on a milkweed flower was recorded and classified into one of ten insect morphogroups (bumblebees, large bees, medium bees, small bees, honeybees, lepidopterans, flies, bugs/beetles, wasps, and ants; Table A3). Surveys were conducted by one individual walking at a steady pace around the entire population in a pre‐determined route. During each visit, the number of inflorescences in the entire population was recorded. Visitation rate was calculated for each survey by dividing the total number of insect visitors by the time surveyed (minutes) and the number of inflorescences present (visits minute^−1^ inflorescence^−1^). Visitation rates were also calculated for each individual morphogroup during each survey.

### 2.5 Pollen removal and deposition

Between five to 50 plants (depending on population size) were numbered and marked in each population. On each marked plant, five random flowers were marked with tape (Brantovision) wrapped discreetly around the pedicel. Marked flowers were monitored throughout the flowering season, and during each site visit, the number of pollinaria removed and the number of pollinia deposited on each marked flower were recorded with the aid of a hand lens (e.g., La Rosa & Conner, 2017).

Pollen removal for each plant surveyed was calculated by summing the number of pollinaria removed across all marked flowers on a given plant and dividing by 25 (total number of available pollinaria in all five flowers), resulting in the proportion of pollinaria removed for each plant. Pollen deposition was calculated by summing the number of pollinia that were inserted on all flowers on a given plant, and dividing by five (number of flowers surveyed per plant), resulting in the mean number of pollinia deposited for each plant. In rare cases, fewer than five flowers were surveyed on a plant (e.g., loss of marked flowers), in which case calculations were adjusted accordingly.

### 2.6 Statistical analyses

(Dis)similarities between developed and undeveloped sites in insect community composition, estimated based on morphogroup visitation rates, were calculated using the Bray–Curtis similarity index. Differences were then visualized using non‐metric multidimensional scaling (NMDS) with the vegan package in R (Oksanen et al., 2022). Between site differences in community composition and visitation rate were analyzed with a permutational multivariate analysis of variance (PERMANOVA), with the ‘adonis2’ function and 10,000 permutations. The contribution of each pollinator morphogroup to differences between developed and undeveloped sites was determined with a similarity percentage (SIMPER) analysis using the vegan package. Morphogroup visitation rates were square root transformed prior to analysis.

Additionally, three mixed effect models were constructed to test for differences in each response variable (visitation rate, pollen removal, and pollen deposition) between developed and undeveloped sites. In each model, year surveyed (2021 or 2022) and site ID were included as random factors. Survey date was included as a random factor for the visitation rate model only. Visitation rate was analyzed using Gamma residual distributions, while pollen removal was analyzed using a Gaussian residual distribution. No residual distribution conformed with the pollen deposition data, so this was transformed (√(“Pollen Deposition”) “+1”) to meet assumptions of normality and a Gaussian residual distribution was used. All models were constructed using the lme4 package (Bates et al., 2022) in R.

## 3 RESULTS

In total, 3045 minutes of insect surveys were conducted across all sites, in which 15,875 total insect visits were recorded from >70 different species across five insect orders. Across the 12 sites, 1205 flowers from 244 plants were surveyed for pollen removal and deposition. On average, 33.6% (0.336 *±* 0.012) of pollinaria were removed and 0.12 ± 0.01 pollinia were deposited per flower. Overall, there was significant site‐wise variation in pollinator visitation rate and pollen transfer (Table A4).

**FIGURE 3:**
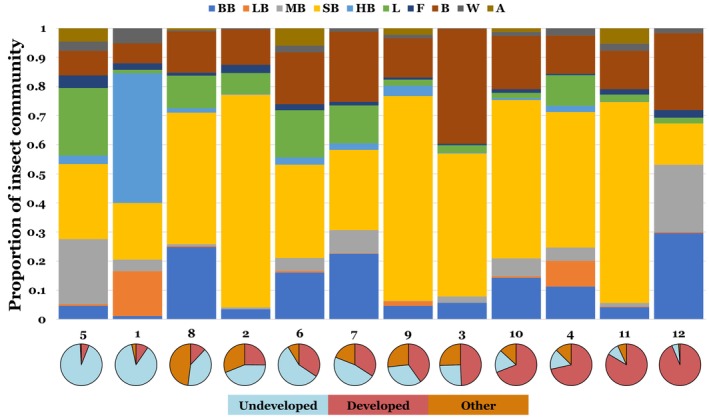
Community composition of milkweed floral visitors observed at the twelve sites. The morphogroups are as follows: bumblebees (BB), large bees (LB), medium bees (MB), small bees (SB), honeybees (HB), lepidopterans (L), flies (F), bugs/beetles (B), wasps (W), and ants (A). The percent land‐use for each site is shown in pie charts below the graph. Other land‐use consists of pastureland and cultivated crops (i.e., agricultural). The first six sites (1–2 and 5–8) were considered undeveloped, and the last six (3–4 and 9–12) were considered developed.

We also observed large site‐wise variation in insect community composition (Figure 3). At 10 of the sites, small bees formed the majority of visits and were the most abundant morphogroup overall, accounting for over half of the 15,875 total visits (52.0%; Figure 3). The five bee morphogroups combined to form 70.7% of all insect visits, with Hymenoptera (bees, wasps, and ants) accounting for 74.7% of visits. Honeybees were most abundant at site 1, but were only present in small numbers at all other sites (Figure 3). Bumblebees were the most abundant visitors at site 12 and were the third most abundant visitor overall, after small bees and bugs/beetles (Figure 3).

**FIGURE 4:**
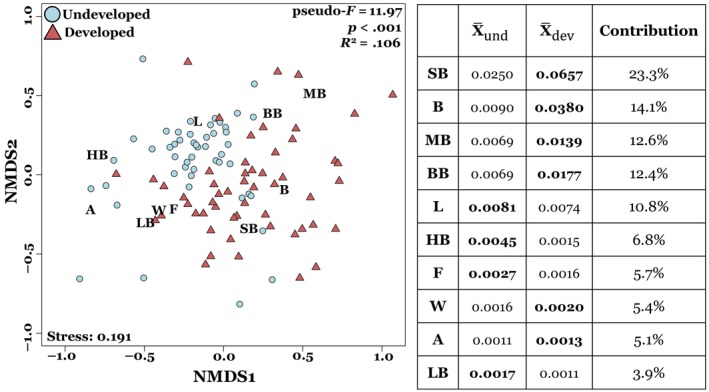
Results from NMDS evaluating differences in milkweed floral visitor composition between developed and undeveloped sites based of visitation rates from all different floral visitor morphogroups. Blue circles represent data from undeveloped sites, while red triangles represent data from developed sites. The table on the right shows the results from the SIMPER analysis. The two columns on the left show the mean visitation rates of the morphogroups at developed and undeveloped sites, with the larger visitation rate in bold. The contribution column expresses the percent contribution of that morphogroup to observed dissimilarities between developed and undeveloped sites. The morphogroups are as follows: small bees (SB), bugs/beetles (B), medium bees (MB), bumblebees (BB), lepidopterans (L), honeybees (HB), flies (F), wasps (W), ants (A), and large bees (LB).

Comparisons of insect community composition based on insect morphogroup visitation rates revealed differences between developed and undeveloped sites (Figure 4). A PERMANOVA analysis showed significant differences in insect community composition between land‐use types (*p* < .001), suggesting that land‐use changes alters the abundance of individual insect morphogroups visiting common milkweed. However, the total explained variance (*R* ^2^) was relatively small (.106), suggesting that land‐use type alone does not fully explain differences in insect community composition. Year and individual site difference also contributed to variation in insect community composition (*p* < .001 for both). SIMPER analysis showed differences in pollinator community composition between developed and undeveloped sites were mainly driven by an increase in the abundance of small bees (23.3% of differences) and bugs/beetles (14.1% of differences) at the more developed sites (Figure 4).

**FIGURE 5:**
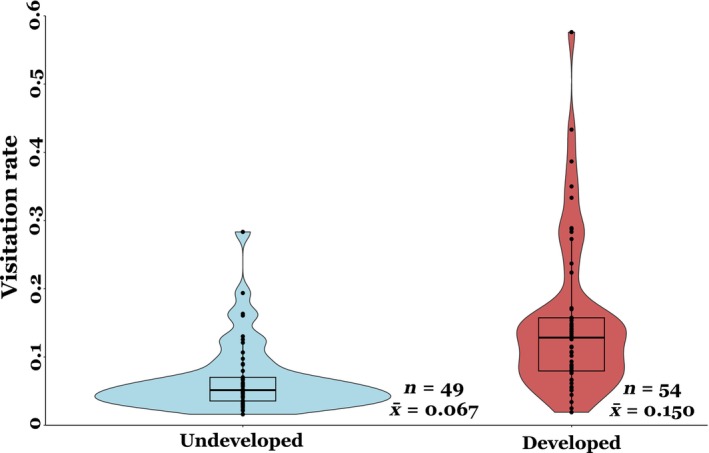
Mean insect floral visitation rate (visits/flower/minute) at developed and undeveloped sites.

**FIGURE 6:**
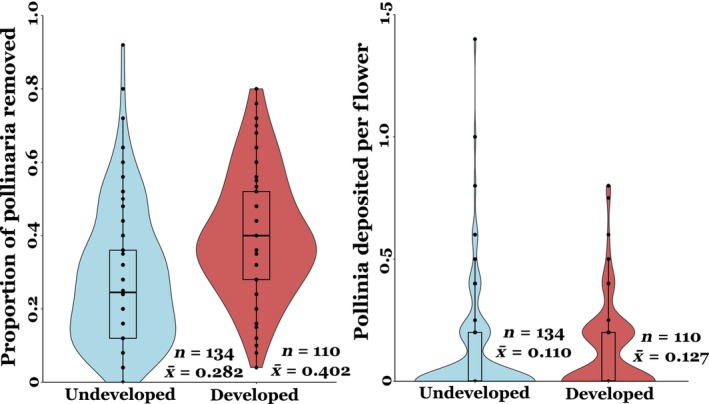
Mean proportion of pollinaria removed and pollinia deposited on milkweed flowers at developed and undeveloped sites.

Mean pollinator visitation rate at the developed sites was more than double compared to that of undeveloped sites (0.150 vs. 0.067, Figure 5), which represents a statistically significant difference (*t* = −2.3, *p* = .01). The proportion of pollinaria removed per flower was significantly higher at the developed sites compared to the undeveloped sites (*t* = −2.04, *p* = .04, Figure 6), but differences in average pollen deposition (pollinaria inserted per flower) between developed and undeveloped sites were not significant (*t* = −0.03, *p* = .9).

## 4 DISCUSSION

Our results show land‐use change can differentially impact the varied components of the pollination process (visitation rate, pollen removal, and pollen deposition), even for a generalist pollinated plant species well‐adapted to developed landscapes. As predicted developed and undeveloped sites differed in the composition of insect morphogropus visiting milkweed flowers. Specifically, there was a higher abundance of small bees in developed areas. Interestingly, however, insect visitation rate and pollen removal were significantly higher in developed areas, while pollen deposition was unimpacted by land‐use change.

Insect visitation rate more than doubled at developed sites compared to undeveloped sites (Figure 5). Among the six developed sites, only two sites had greater than 10% “Developed, High Intensity” land within a 2 km radius, while no site had greater than 20% “Developed, High Intensity” (per NLCD land‐use definitions, see methods and Table A2). Additionally, no site had greater than 50% combined “Developed, High Intensity” and “Developed, Medium Intensity,” and the site with highest percent of developed land‐use was in a large community garden with abundant floral resources and nesting area (non‐impervious surfaces) (Table A2). Thus, while all study sites exhibit varying levels of moderate land‐use change, no site occurred in a heavily developed environment. Although our study was not specifically designed to evaluate the effects of different intensity levels our disturbance, our results lend general support to the growing evidence suggesting increased pollinator abundance in moderately disturbed areas (Biella et al., 2022; Carper et al., 2014; Sing et al., 2016; Theodorou et al., 2016; Udy et al., 2020; Wenzel et al., 2020). Furthermore, when we analyzed how the different components of pollination success evaluated here change with increasing percent of undeveloped habitat across all of our sites we found a negative effect on visitation rate only (*p* < .05; Table A5), suggesting that insect visitation rate decreases with increasing percent of undeveloped land. Other studies have also suggested that moderate levels of disturbance should promote higher pollinator species richness and abundance by creating a heterogeneous habitat landscape that allows for the coexistence of pollinators that typically occur in natural areas and those that can take advantage of urbanized habitats (Banaszak‐Cibicka et al., 2018). Furthermore, when evaluating the effects of land‐use change it is also important to consider the spatial patterning of change leading to varying degrees of habitat heterogeneity (Lander et al., 2013). For instance, past studies have found that pollinator richness decreases in undeveloped areas when isolated from one another within a developed landscape, further emphasizing the importance of habitat connectivity in positively maintaining ecosystem function (Aizen & Feinsinger, 1994; Jauker et al., 2009). In our study, milkweed populations existed in a highly heterogeneous landscape of development, with each developed milkweed population within 2 km of at least one urban garden or park, suggesting each site was sufficiently connected to undeveloped areas. Overall, this study emphasizes the need for more detailed studies that further consider the intensity and pattern of land‐use change to fully understand the complexity of its effects on ecological processes, such as pollination.

Pollen removal also increased at more developed sites, likely due to an overall increase in insect visitation rates (Figure 5; also see Harder, 1990; Rush et al., 1995). Interestingly, higher pollen removal did not translate to higher rates of pollen deposition on milkweed flowers. This pattern may be due to the differences in floral visitor composition between developed and undeveloped sites, specifically the increase in bugs/beetles and small bees in more developed areas. There was an over fourfold increase in bug/beetle visitation rate in developed areas. Among all the bugs/beetles observed, however, only soldier beetles (Cantharidae) were frequently observed with attached pollinaria, suggesting soldier beetles were the only beetles that effectively pollinated common milkweed in this study (Kephart & Theiss, 2004). Hemipterans (true bugs) have widely been ignored as milkweed pollinators in past studies. During our insect surveys, two Hemipteran genera were observed, one of which, *Oncopeltus*, was never observed with attached pollinaria, as found in past studies (Fishbein & Venable, 1996). However, the genus *Euschistus* was occasionally observed with attached pollinaria, suggesting they may be capable of pollinating common milkweed. Further studies are needed to expand the current knowledge on the effectiveness of Hemipterans as common milkweed pollinators, although in this study they likely had a negligible contribution to both pollen removal and deposition due to their low overall abundance. Thus, differences in pollen removal and deposition between developed and undeveloped sites are likely caused by the over twofold increase in small bee visitation in developed areas. Small bees are functionally able to remove pollinaria and were frequently observed with attached milkweed pollinaria in this study. Although the weight of the attached pollinia may disproportionally limit their movement, hence increasing the risk of predation and decreasing their ability to successfully deposit milkweed pollinia once removing the pollinaria (Morse, 1985; Morse & Fritz, 1989). Thus, the high abundance of small bees in developed areas may have resulted in an increase in pollen removal, but not in an associated increase in pollen deposition. It is also possible that floral visitors may differ in their flower‐handling or grooming behavior between developed and undeveloped areas leading to differences in active or passive loss of pollinaria between visits. However, we do not have any observational evidence to suggest that this would be the case in this study. Overall, more detailed studies on the pollination efficiency (pollen removal and deposition) of each pollinator group are needed, specifically to determine whether different pollinator groups exhibit different behaviors which may influence their effectiveness at depositing milkweed pollinia at developed compared to undeveloped areas. So far, the few studies on milkweed pollination that have included bugs, beetles, and small bees have suggested that they are inefficient milkweed pollinators (with the notable exception of soldier beetles) (Fishbein & Venable, 1996; Kephart & Theiss, 2004). Moreover, when small bees and bugs/beetles were removed from our analysis, differences in insect visitation rate were no longer statistically significant between developed and undeveloped sites (*p* = .6), further indicating that an increase in overall insect abundance or visitation rate does not necessarily lead to increased pollination success. Additionally, preliminary data on milkweed reproductive output (fruit and seed production) showed no difference between developed and undeveloped sites (Figure A1), suggesting a disconnect between increased insect visitation and reproductive success. Fruit and seed production for instance have been commonly shown to strongly respond to variation in resource availability, which likely varies across the landscape. Overall, our study highlights the importance of considering pollen removal and deposition simultaneously, as they can be differentially impacted by changes in land‐use.

Our results stress the importance of evaluating the impacts of land‐use changes at multiple steps in the pollination process. Studies that focus on a single component of pollination, especially those that only look at pollinator visitation, likely provide an incomplete assessment on the effects of land‐use changes on pollination success. Our findings suggest that the effects of land‐use changes on pollination can range from positive to negative, and likely depend on the intensity of disturbance. While not all aspects of pollination success were positively impacted by land‐use change, none were negatively impacted. Perhaps suggesting that moderately developed areas have the potential to not only maintain, but in some cases promote the health of vital ecosystem services. This result stresses the importance of considering and managing urban areas not as ecologically dead zones, but as potential novel habitats where key aspects of biodiversity and ecological interactions can be maintained.

## AUTHOR CONTRIBUTIONS

**David J. Rockow:** Conceptualization (equal); data curation (lead); formal analysis (lead); investigation (lead); methodology (lead); visualization (equal); writing – original draft (lead). **Carlos Martel:** Data curation (supporting); formal analysis (equal); investigation (supporting); visualization (equal); writing – review and editing (equal). **Gerardo Arceo‐Gómez:** Conceptualization (equal); project administration (equal); resources (equal); supervision (equal); writing – review and editing (equal).

## CONFLICT OF INTEREST STATEMENT

The authors have no conflicts of interest to declare.

## Supporting information


Appendix S1.

## Data Availability

All data will be placed in an open digital repository (Dryad) upon acceptance DOI: 10.5061/dryad.n5tb2rc3c. Private for peer review link: https://datadryad.org/stash/share/nMolq4AoMba6fn9XBiglfeFkzi1qNNb1ZO_MlidMQMQ.
