# Supplementary material for: Differential impacts of land‐use change on multiple components of common milkweed (Asclepias syriaca) pollination success
Source: Ecol Evol. 2024 Jun 6;14(6):e11494. doi: 10.1002/ece3.11494 (PMC11156956; doi:10.1002/ece3.11494)
Supplement: Supplementary file 1 — Appendix S1. [file ECE3-14-e11494-s001.zip › Appendix.docx]

**Appendix:**

Table A1: The location, year surveyed, number of flowers surveyed/number of plants surveyed, total number of flowering plants, and insect surveying time (total) for all study sites.

| **Site** | **Location** | **Year Surveyed** | **Flowers / Plants Surveyed** | **Total Plants in Population** | **Approximate Population Area (m^2^)** | **Total Insect Surveying Time (min)** |
| --- | --- | --- | --- | --- | --- | --- |
| **1** | **36° 19' 13.43”N,**  **-82° 2' 40.92”W** | **2021** | **24/6** | **15** | **75** | **120** |
| **2** | **36° 30' 14.40”N,**  **-82° 28' 4.80”W** | **2021** | **95/20** | **185** | **343** | **150** |
| **3** | **36° 23' 4.92”N,**  **-82° 23’ 6.00”W** | **2021** | **95/20** | **35** | **78** | **240** |
| **4** | **36° 18' 57.96”N,**  **-82° 19' 28.20” W** | **2021** | **97/20** | **73** | **340** | **390** |
| **5** | **36° 10' 42.96”N,**  **-82° 6' 44.28”W** | **2022** | **130/26** | **71** | **287** | **435** |
| **6** | **36° 33' 40.32”N,**  **-82° 13’ 21.00”W** | **2022** | **150/30** | **189** | **228** | **300** |
| **7** | **36° 30' 9.71”N,**  **-82° 28' 57.71”W** | **2022** | **140/28** | **102** | **304** | **270** |
| **8** | **36° 26' 9.96”N,**  **-82° 22' 13.80”W** | **2022** | **125/25** | **162** | **328** | **240** |
| **9** | **36° 20' 34.80”N,**  **-82° 15' 17.64”W** | **2022** | **100/20** | **614** | **276** | **240** |
| **10** | **36° 21' 50.76”N,**  **-82° 23' 46.68"W** | **2022** | **75/15** | **168** | **152** | **195** |
| **11** | **36° 18' 5.76”N,**  **-82° 21' 32.40”W** | **2022** | **125/25** | **124** | **50** | **300** |
| **12** | **36° 18' 44.28”N,**  **-82° 20' 56.04”W** | **2022** | **50/10** | **16** | **14** | **165** |

Table A2: The table on the left shows the percent land-use of each site for the NLCD land-use categories, excluding open water. The four NLCD developed categories are defined as follows: developed open space has <20% impervious surface, developed low intensity has between 20-49% impervious surface, developed medium intensity has between 50-79% impervious surface, and developed high intensity has >80% impervious surface. Percent land-use for each site using all the 15 NLCD land-use categories. Different colors show categories assign to undeveloped, developed, and agricultural habitat.

Table A3: Representative genera for the insect morphogroups. All insects that landed on a milkweed flower were recorded. Thus, not all of the recorded visitors are necessarily pollinators.

| **Morphogroup** | **Observed Genera** |
| --- | --- |
| **Bumblebees**  **(BB)** | *Bombus* |
| **Large Bees**  **(LB)** | *Megachile*, *Xylocopa* |
| **Medium Bees (MB)** | *Andrena, Coelioxys, Heriades*, *Lasioglossum, Megachile, Peponapis* |
| **Small Bees**  **(SB)** | *Augochlora*, *Ceratina*, *Halictus*, *Hylaeus,* *Lasioglossum* |
| **Honeybees**  **(HB)** | *Apis* |
| **Lepidopterans**  **(L)** | *Atalopedes*, *Atteva*, *Battus*, *Chlosyne*, *Cisseps*, *Colias*, *Cupido, Cycnia*, *Danaus*, *Eichlinia*, *Epargyreus*, *Eurytides*, *Geina*, *Harrisina*, *Hemaris*, *Macaria*, *Papilio*, *Phyciodes*, *Satyrium*, *Spragueia*, *Synanthedon*, *Thyris* |
| **Flies**  **(F)** | *Geron*, *Juriniopsis*, *Physocephala*, *Sphaerophoria*, *Trichopoda* |
| **Beetles & Bugs**  **(B)** | *Chauliognathus*, *Cotinis*, *Euphoria*, *Euschistus*, *Oncopeltus*, *Photinus*, *Popillia*, *Stiretrus*, *Tetraopes*, *Typocerus* |
| **Wasps**  **(W)** | *Ammophila*, *Cerceris*, *Dielis*, *Dolichovespula*, *Polistes*, *Scolia*, *Sphex* |
| **Ants**  **(A)** | *Camponotus*, *Formica* |

Table A4: Insect floral visitation rate, proportion of pollinaria removed and pollinaria deposition values for each study site..

| **Site** | **Visitation Rate** | **Proportion of pollinaria Removed** | **Pollinaria Deposition** |
| --- | --- | --- | --- |
| **1** | 0.113 | 0.308 | 0.208 |
| **2** | 0.198 | 0.496 | 0.263 |
| **3** | 0.289 | 0.476 | 0.211 |
| **4** | 0.124 | 0.394 | 0.072 |
| **5** | 0.053 | 0.256 | 0.062 |
| **6** | 0.053 | 0.182 | 0.060 |
| **7** | 0.053 | 0.202 | 0.079 |
| **8** | 0.051 | 0.338 | 0.112 |
| **9** | 0.065 | 0.326 | 0.100 |
| **10** | 0.124 | 0.406 | 0.093 |
| **11** | 0.098 | 0.444 | 0.168 |
| **12** | 0.209 | 0.300 | 0.060 |

Table A5: Results (*P*-values) of GLMs using percent of undeveloped land-use type as the explanatory variable to evaluate its effects on insect floral visitation rate, pollinaria removal and pollen deposition.

|  | % Undeveloped |
| --- | --- |
| Visitation Rate | 0.029* |
| Pollinaria Removal | 0.496 |
| Pollinaria Deposition | 0.451 |

Figure A1. Fruit & seed production boxplots. Data from the undeveloped populations are shown to the left in green, while data from the developed populations is shown in blue on the right. Fruit data is from each plant surveyed, while seed data is from each fruit collected.
