# Supplementary material for: Differential impacts of land‐use change on multiple components of common milkweed (Asclepias syriaca) pollination success
Source: Ecol Evol. 2024 Jun 6;14(6):e11494. doi: 10.1002/ece3.11494 (PMC11156956; doi:10.1002/ece3.11494)
Supplement: Supplementary file 1 — Appendix S1. [file ECE3-14-e11494-s001.zip › tableA2.docx]

**Agricultural**

**Undeveloped**

**Developed**

| **Site** | **% Developed**  **Open Space** | **% Developed**  **Low Intensity** | **% Developed Medium Intensity** | **% Developed**  **High Intensity** | **% Developed**  **Total** | **% Barren Land** | **% Deciduous Forest** | **% Evergreen Forest** | **% Mixed Forest** | **% Shrub** | **% Grassland** | **% Woody Wetlands** | **% Herbaceous Wetlands** | **% Undeveloped Total** | **% Pastureland** | **% Cultivated**  **Crops** | **% Agricultural Total** |
| --- | --- | --- | --- | --- | --- | --- | --- | --- | --- | --- | --- | --- | --- | --- | --- | --- | --- |
| **1** | 7.95 | 1.11 | 0.43 | 0.09 | **9.58** | 0.10 | 45.36 | 4.22 | 36.28 | 0.53 | 0.57 | 0 | 0 | **87.05** | 3.37 | 0 | **3.37** |
| **2** | 15.72 | 7.48 | 1.82 | 0.11 | **25.13** | 0.10 | 39.87 | 0.64 | 2.63 | 0.21 | 0.34 | 0 | 0 | **43.80** | 31.07 | 0 | **31.07** |
| **3** | 17.87 | 20.73 | 7.92 | 2.81 | **49.33** | 0.27 | 19.37 | 0.08 | 2.46 | 0.83 | 2.13 | 0 | 0 | **25.14** | 25.48 | 0.05 | **25.53** |
| **4** | 24.91 | 26.28 | 13.63 | 6.71 | **71.52** | 0.11 | 13.93 | 0.02 | 0.74 | 0.36 | 0.62 | 0.05 | 0 | **15.82** | 12.66 | 0 | **12.66** |
| **5** | 5.92 | 0.08 | 0.01 | 0 | **6.01** | 0 | 73.25 | 0.45 | 19.25 | 0.06 | 0.14 | 0 | 0 | **93.15** | 0.85 | 0 | **0.85** |
| **6** | 16.35 | 10.91 | 5.01 | 2.16 | **34.43** | 0 | 52.34 | 0.70 | 3.13 | 0.02 | 0.17 | 0.39 | 0.04 | **56.80** | 8.77 | 0 | **8.77** |
| **7** | 21.37 | 10.43 | 2.24 | 0.12 | **34.15** | 0.08 | 42.94 | 0.63 | 2.75 | 0.08 | 0.30 | 0 | 0 | **46.77** | 19.08 | 0 | **19.08** |
| **8** | 7.04 | 4.04 | 0.89 | 0.07 | **12.04** | 0.72 | 31.71 | 0.29 | 4.47 | 0.72 | 2.10 | 0 | 0 | **40.01** | 47.31 | 0.64 | **47.95** |
| **9** | 12.40 | 13.58 | 8.86 | 5.39 | **40.24** | 0.01 | 28.76 | 0.23 | 2.85 | 0 | 0.21 | 1.02 | 0 | **33.08** | 26.68 | 0 | **26.68** |
| **10** | 19.76 | 24.28 | 15.96 | 9.05 | **69.05** | 0.06 | 14.49 | 0.04 | 1.83 | 1.11 | 0.19 | 0.01 | 0 | **17.73** | 13.22 | 0 | **13.22** |
| **11** | 24.48 | 26.56 | 21.40 | 11.10 | **83.54** | 0 | 8.24 | 0.06 | 1.05 | 0.03 | 0.19 | 0 | 0 | **9.56** | 6.90 | 0 | **6.90** |
| **12** | 18.25 | 33.27 | 26.83 | 15.29 | **93.64** | 0.06 | 4.61 | 0.02 | 0.23 | 0 | 0.27 | 0 | 0 | **5.19** | 1.18 | 0 | **1.18** |
